# The axonal cytoskeleton: from organization to function

**DOI:** 10.3389/fnmol.2015.00044

**Published:** 2015-08-14

**Authors:** Josta T. Kevenaar, Casper C. Hoogenraad

**Affiliations:** Cell Biology, Faculty of Science, Utrecht UniversityUtrecht, Netherlands

**Keywords:** axon, cytoskeleton, actin, microtubule, kinesin, transport, presynapse, axon initial segment

## Abstract

The axon is the single long fiber that extends from the neuron and transmits electrical signals away from the cell body. The neuronal cytoskeleton, composed of microtubules (MTs), actin filaments and neurofilaments, is not only required for axon formation and axonal transport but also provides the structural basis for several specialized axonal structures, such as the axon initial segment (AIS) and presynaptic boutons. Emerging evidence suggest that the unique cytoskeleton organization in the axon is essential for its structure and integrity. In addition, the increasing number of neurodevelopmental and neurodegenerative diseases linked to defect in actin- and microtubule-dependent processes emphasizes the importance of a properly regulated cytoskeleton for normal axonal functioning. Here, we provide an overview of the current understanding of actin and microtubule organization within the axon and discuss models for the functional role of the cytoskeleton at specialized axonal structures.

## Introduction

**Figure 1:**
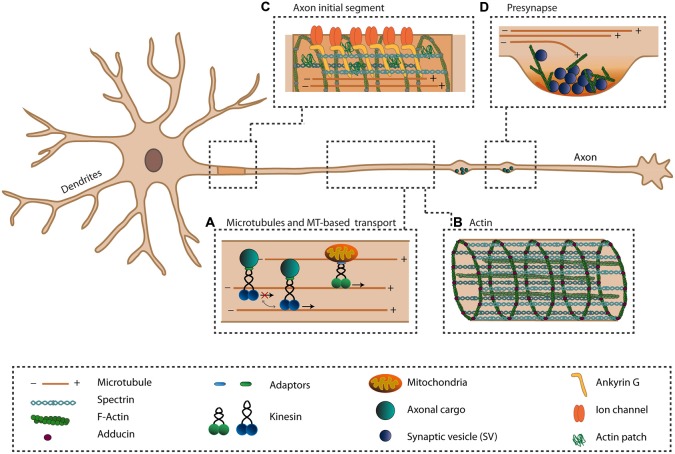
**The axonal cytoskeleton and axon-specific structures**. The axon is specialized in transmitting information to other cells. To ensure this function, the axon has a unique cytoskeletal organization **(A,B)** and has several specialized structures **(C,D)**. **(A)** The unique unipolar orientation of the microtubules (MTs) within the axon provides anterograde transports of various axonal cargoes via plus-end directed kinesins. Various mechanisms exist that regulate the activity of kinesins. **(B)** The actin cytoskeleton within the axon exists as periodically spaced rings underneath the axonal plasma membrane, organized by spectrin and adducing, and provides the axon with elastic and stable support. Along the axon, bundles of actin are present. **(C)** The axon initial segment (AIS) is important for the initiation of action potentials and maintaining neuronal polarization by acting as a transport filter. Within the AIS, a dense meshwork of cytoskeletal and scaffolding proteins exists where Ankyrin-G (AnkG) links transmembrane proteins to the actin and microtubule cytoskeleton. **(D)** At the presynaptic site, neurotransmitter-filled synaptic vesicles (SVs) are docked at the presynaptic membrane and undergo exocytosis upon the arrival of an action potential. Within the presynapse, actin is proposed to exist in a branched network where it may function in the controlling exo- and endocytosis, recruiting and positioning of SVs and organizing the active zone (AZ).

Neurons are the basic cells that process information within the brain. They are compartmentalized into two morphologically, molecularly and functionally distinct domains; the axonal and the somatodendritic compartments. Multiple short and highly branched dendrites function in receiving and integrating electrical synaptic inputs from thousands of neurons. In contrast, only a single axon is responsible for transmitting this integrated information in the form of an action potential, an electrical excitation wave that travels along the axonal membrane. To ensure that information is transmitted properly, the axon has a unique cytoskeletal organization and contains several specialized structures, including the axon initial segment (AIS) and presynaptic boutons (Figure 1).

The typical morphology and specialization of the axon is formed during a number of distinct developmental stages. Initially, neurons develop several short processes, called neurites. Then, upon neuronal polarization, one of these neurites grows longer than the others and becomes the axon, whereas the other neurites are destined to become the dendrites (Dotti et al., 1988). Axon specification is regarded as the fundamental process that gives the neuron its polarized morphology and segregates its neuronal functions into the somatodendritic and axonal compartments (Stiess and Bradke, 2011). Shortly after the initial specification of the axon, the AIS assembles through the local accumulation of specific proteins at the proximal part of the axon which starts around 4 days in culture and continues with 8 more days (Boiko et al., 2007; Hedstrom et al., 2007). Through the assembly of the AIS, the axon is separated from the rest of the neuron to maintain neuronal polarity (Leterrier and Dargent, 2014). Later on, numerous signaling molecules direct further axonal outgrowth, the formation of synaptic contacts and subsequently, presynaptic differentiation through the assembly of presynaptic protein complexes (Chia et al., 2013).

The neuronal cytoskeleton, which is composed of microtubules (MTs), actin filaments and neurofilaments, enacts important functions in both the establishment and maintenance of neuronal polarity, morphology and integrity of axons (Luo, 2002; Barnes and Polleux, 2009; Kapitein and Hoogenraad, 2011). The main function of neurofilaments, which are particularly abundant in axons, is to control the axon diameter and thereby axonal conductance (Yuan et al., 2012). MTs and actin filaments mainly affect axon specification and growth and provide the roads for long- and short-range active axonal transport (Luo, 2002; Kapitein and Hoogenraad, 2011; Sainath and Gallo, 2015). Both MTs and actin filaments are dynamic structures, meaning that they continuously grow and shrink, which facilitates the continuous remodeling of the cytoskeleton. Already during the initial stages of neuronal development and neurite outgrowth, the cytoskeleton enacts an important role in generating intracellular forces and acts as a signaling device (Witte and Bradke, 2008). It is suggested that neurite initiation and outgrowth depends on the local increase in actin dynamics in combination with microtubule stabilization (Flynn et al., 2012). For example, during neuronal polarization, actin waves enhance the delivery of actin and actin-associated proteins to the putative axon which promotes axonal elongation (Flynn et al., 2009), which is mediated by the directional assembly and disassembly of membrane-anchored F-actin (Katsuno et al., 2015). In addition, kinesin-1 driven sliding of MTs may provide the force needed for initial neurite outgrowth (Lu et al., 2013). During the later processes of axonal outgrowth, microtubule and actin dynamics are critical for driving growth cone motility and axon guidance (Kolodkin and Tessier-Lavigne, 2011; Vitriol and Zheng, 2012; Gomez and Letourneau, 2014; Liu and Dwyer, 2014), but since excellent recent reviews already exist on this topic their role in these processes will not be discussed here. Finally, when contacts with other neurons are made, actin rearrangements play important roles in proper development of presynaptic sites through the organization of numerous presynaptic components (Cingolani and Goda, 2008; Nelson et al., 2013).

Since MTs and actin filaments are involved in various stages of axon formation and outgrowth, they have recently received much attention in studies on axon regeneration. These studies are all aimed at increasing the regenerative properties of central nervous system (CNS) axons. This could be achieved by affecting cytoskeletal rearrangements directly (Gordon-Weeks and Fournier, 2014) or by increasing axonal transport of receptors that mediate growth by signaling onto the cytoskeleton in response to extracellular cues (Eva and Fawcett, 2014). For example, microtubule stabilization after injury has been shown to promote axon regeneration *in vivo* (Hellal et al., 2011). Also, a more recent study demonstrated the potency of the Food and Drug Administration (FDA) approved microtubule-stabilizing drug epothilione B in promoting axon growth and functional recovery after CNS injury in rodent spinal cords (Ruschel et al., 2015). In addition, the developmentally regulated exclusion of growth-related receptors from the axon is suggested to account for the lack of regenerative ability of mature CNS axons. This regenerative ability was demonstrated to be restored by altering axonal trafficking to reintroduce these growth-related proteins into the axon *in vitro* (Franssen et al., 2015). Moreover, kinesin-1 mediated microtubule sliding has also been demonstrated to be involved in axonal regeneration in *Drosophila* (Lu et al., 2015). Normally, microtubule sliding is developmentally down-regulated, but injury-induced calcium influx induces local microtubule disassembly and subsequently the formation of local microtubule arrays with mixed polarity (del Castillo et al., 2015; Lu et al., 2015). The formation of these mixed microtubule polarity arrays re-introduces the ability for kinesin-1 mediated microtubule sliding and thereby neurite outgrowth (Lu et al., 2015). In general, severe cytoskeletal rearrangements occur after injury and targeted cytoskeletal rearrangement may be a promising strategy for enhancing axon regeneration.

In addition to axon regeneration, the axonal cytoskeleton has also gained much attention in respect to its association with several neurological diseases. For example, several developmental and neurological disorders have been described in which defects in axonal transport, outgrowth, targeting and synapse functioning are caused by disruption of axonal cytoskeleton-dependent processes (De Vos et al., 2008; Letourneau, 2009; Franker and Hoogenraad, 2013; Breuss and Keays, 2014). For example, the motor neuron degenerative disease Amyotrophic Lateral Sclerosis (ALS) has been associated with axonal cytoskeletal rearrangements and axonal transport dysfunction (Robberecht and Philips, 2013). In Alzheimer’s disease (AD), dissociation and mis-sorting of the axonal microtubule-associated protein tau and cytoskeletal disruptions are linked to transport deficits and synaptic dysfunction (Zempel and Mandelkow, 2014). In addition, impaired regulation of microtubule stability, caused by spartin deficiency, is suggested to affect presynaptic development and axonal survival which underlies the neurodegenerative disease Troyer syndrome hereditary spastic paraplegia (HSP; Nahm et al., 2013). These neurological disorders emphasize the importance of a functional and properly regulated axonal cytoskeleton for normal axonal functioning. In this review, we will give an overview of specialized axonal structures and relate their specific functions to the unique organization of the axonal cytoskeleton.

### Actin and Microtubule Organization Within the Axon

MTs are cylindrical polymers built up from α- and β-tubulin hetrodimers, with a fast-growing plus-end and a more stable minus-end. These tubulin polymers switch stochastically between polymerization and depolymerization, a process called dynamic instability (Mitchison and Kirschner, 1984). The dynamics of MTs are regulated by a large number of factors, including microtubule-associated proteins (MAPs), motor proteins, post-translational tubulin modifications and plus-end tracking proteins (Schuyler and Pellman, 2001; Dehmelt and Halpain, 2005; Akhmanova and Steinmetz, 2010; Janke and Kneussel, 2010; Drummond, 2011; Niwa, 2015). For example, various MAPs that decorate axonal MTs, including MAP1B and the axon-specific protein tau, influence microtubule dynamics by stabilization. (Cleveland et al., 1977; Drechsel et al., 1992; Tortosa et al., 2013; Derisbourg et al., 2015). In axons, MTs form an unique unipolar organization, where all MTs are oriented with their plus-end towards the axon tip, whereas in dendrites their orientation is mixed (Baas et al., 1988; Stepanova et al., 2003; Stone et al., 2008; Kapitein et al., 2010; Figure 1A). This unique orientation in the axon has important implications for its function, since it affects the specific sorting of axonal and dendritic cargos (Kapitein and Hoogenraad, 2011).

Actin filaments (F-actin), polymers built up from globular actin (G-actin), are polarized due to the orientation of each actin monomer in the filament. On the growing, barbed end, subunits are added while on the opposite side, the pointed end, monomers dissociate (Letourneau, 2009). Due to the weak interaction between these actin monomers, actin filaments rapidly shift between polymerization and depolymerization states. Actin dynamics is regulated by numerous actin-binding proteins (ABPs) via various mechanisms. For instance, ABPs can act by sequestering G-actin, nucleating actin filaments, capping or binding the barbed or pointed end to inhibit or promote polymerization or depolymerization respectively, severing of actin filaments, bundling, crosslinking, stabilizing, and anchoring of F-actin to other cellular components (Letourneau, 2009). F-actin is important for organizing the plasma membrane and for providing a cortical scaffold for the localization of protein complexes (Letourneau, 2009). In synapses, for example, stable F-actin plays a scaffolding role while rapid reorganization of actin remodels the synaptic structure during neuronal plasticity (Cingolani and Goda, 2008). Due to its difficulty to visualize, the organization of axonal actin has been more challenging to characterize precisely. For long, actin was assumed to exist only in a subaxolemmal space that contains a dense network of thin actin filaments, which connect the plasma membrane to the central microtubule cytoskeletal network (Hirokawa, 1982). Using electron microscopy techniques, patches of branched actin filaments, organized in the form of meshworks, have been identified along the axon. Distally, these patches contribute to the formation of axonal filopodia (Korobova and Svitkina, 2010; Spillane et al., 2011; Jones et al., 2014), which give rise to axonal collateral branches (Gallo, 2011). More recently, using super-resolution microscopy, axonal actin was found to be organized in regularly spaced rings that wrapped around the axon beneath the plasma membrane which are spaced and connected by spectrin (Xu et al., 2013; Lukinavičius et al., 2014). These ring-like structures along the axon arise already during axon specification and are formed by short actin filaments that are capped by adducin and are connected by spectrin tetramers that create a periodicity of ~180–190 nm between the rings (Xu et al., 2013; D’Este et al., 2015; Figure 1B). This periodic sub-membrane lattice structure is suggested to provide elastic and stable mechanical support and to organize the molecular organization of the axonal membrane (Xu et al., 2013; D’Este et al., 2015). Besides the subcortical actin organization, bundles of actin are present along the axon where their abundance is highly dependent on the developmental stage (D’Este et al., 2015; Figure 1B). Likely, these actin bundles represent a more dynamic axonal F-actin population as was recently observed using live-cell imaging (Ganguly et al., 2015). Here, trails of actin are suggested to nucleate from specific sites along the axon, likely from the surface of stationary endosomes. These dynamic axonal F-actin filaments were found to be formin, but not Arp2/3, dependent and allow for the rapid availability of actin within the axon (Ganguly et al., 2015). This suggests a dual population of axonal actin, with on the axonal plasma membrane the more stable actin rings, providing mechanical support, and the dynamic inta-axonal actin filaments, providing flexibility needed for maintaining axonal and synaptic plasticity (Ganguly et al., 2015).

### The Role of the Axonal Cytoskeleton at the Axon Initial Segment

One of the unique features of the axon is AIS, localized at the proximal part of the axon. The AIS is rich in ion-channels, scaffolding proteins, cellular adhesion molecules and cytoskeletal proteins, and is essential for the initiation of action potentials and thereby proper functioning of axon (Lai and Jan, 2006; Ogawa and Rasband, 2008). In addition, the AIS plays a key role in maintaining the axonal identity through its specialized cytoskeletal organization, by acting as a diffusion barrier to restrict proteins and lipids to either the axonal or somatodendritic compartment and by acting as a filter for active transport to prevent dendritic cargoes from entering the axon (Leterrier and Dargent, 2014; Yoshimura and Rasband, 2014). Recently, experiments using live-cell imaging of various neuronal cargoes demonstrated that dendritic cargoes abruptly stopped entering the axon, while axonal vesicles passed through the AIS without impediment, further supporting the notion that the AIS functions in actively selecting cargoes (Petersen et al., 2014). This barrier function is supported by a highly specialized protein network localized at the AIS. Here, the scaffolding protein Ankyrin-G (AnkG) links transmembrane proteins and βIV-spectrin to the actin and microtubule cytoskeleton (Grubb and Burrone, 2010; Rasband, 2010; Bennett and Lorenzo, 2013). Together, scaffolding and cytoskeletal proteins set up a dense meshwork underneath the plasma membrane that is essential to build up and maintain a functional AIS (Jones et al., 2014; Figure 1C).

Within the AIS, cytosolic actin has been identified to exist in small networks, very similar to the actin patches observed in the more distal axon segments (Watanabe et al., 2012; D’Este et al., 2015), where individual filaments are thought to be oriented with their plus-end directed towards the cell body (Watanabe et al., 2012). Recent EM experiments demonstrated that actin filaments are rather sparse within the AIS and exhibit no polarized orientation within the AIS (Jones et al., 2014). Regardless of the precise organization of the actin filaments, the presence of actin patches may argue for a model where the AIS functions as a selective transport filter. It has been demonstrated that dendritic cargoes enter both the dendrites and the axon with similar frequencies, but hold their trafficking within the AIS in an actin-dependent manner. In this model, cargoes that have active myosin motors attached are excluded from entering the axon by anchoring to the actin patches (Al-Bassam et al., 2012). The view that actin has a critical function in the AIS is further supported by findings where disruption of the actin filter causes loss of polarized transport of axonal and dendritic cargoes (Winckler et al., 1999; Song et al., 2009). However, most dendritic cargoes have already stopped at the proximal region of the axon, and do not move into the AIS (Nakada et al., 2003; Song et al., 2009). Moreover, polarized transport was observed shortly after axon specification, even before AIS assembly and thus before the appearance of an actin meshwork at the AIS (Petersen et al., 2014). Therefore, these findings question the model of an actin-dependent selective cargo transport filter but may suggest an alternative function for actin patches at the AIS. Since the actin patches co-localize with presynaptic proteins (D’Este et al., 2015), one recent model suggests that they are axonal scaffold sites and play a role in supporting presynaptic boutons (Sankaranarayanan et al., 2003; Waites et al., 2011).

Besides actin, a role of MTs has also been proposed for the AIS filter and for the assembly of the AIS. During neuronal polarization, the first distinct feature of the proximal axon is microtubule bundling. Recently, using electron microscopy techniques, it has been demonstrated that this dense network of microtubule bundles arises even before the assembly of major AIS proteins, including AnkG (Jones et al., 2014). Later, the microtubule bundles acquire an AIS-specific submembranous dense coat that contains a network of various AIS proteins (Jones et al., 2014). The microtubule plus-end binding proteins EB1 and EB3 were identified as proteins that could link MTs to AnkG and stabilize the microtubule lattice in the AIS (Leterrier et al., 2011). Thus, the specialized organization at the AIS physically links AnkG to microtubule bundles and βIV-spectrin to cortical actin, which provides a strong structural basis for the AIS-dependent filter (Figure 1C).

### The Role of Presynaptic Actin

Other unique, highly specialized structures within the axon are the presynaptic sites, which are essential for transmitting information to the connecting neuron. Together with the postsynapse of the receiving neuron, they form the sites where the actual communication between neurons takes place (Sudhof, 2004). The release of neurotransmitters is a critical step in the initiation of synaptic transmission. Neurotransmitter release is caused by exocytosis of synaptic vesicles (SVs) at the presynaptic active zone (AZ), triggered by the arrival of an action potential. At the AZ, the SV cycle underlies the regulated release of neurotransmitters and includes the recruitment, docking and priming of SVs at the presynaptic plasma membrane. This is followed by calcium-triggered exocytosis and subsequent endocytosis of the SV membrane, and concluded by recycling to replenish the SV pool and to sustain the vesicle cycle (Chua et al., 2010; Gundelfinger and Fejtova, 2012; Südhof, 2012). These various functions are executed by several overlapping sets of molecular machineries, which are dynamically assembled into a core macromolecular scaffold within the AZ (Chua, 2014).

Actin was originally thought to be involved in the assembly and development of the presynaptic sites (Zhang and Benson, 2001; Nelson et al., 2013). Findings in *C. elegans* and *Drosophila* indicate a key function of F-actin assembly in the initial stages of synaptogenesis (Chia et al., 2012, 2014; Koch et al., 2014). Actin dynamics, mediated by the Arp2/3 complex, have been shown to be critical for synapse formation in flies (Koch et al., 2014). In *C. elegans*, local F-actin rearrangements, triggered by the interaction of the synaptic cell adhesion molecules SYG-1 and SYG-2, are important for the subsequent recruitment and assembly of AZ proteins, including SYD-2/liprin-α, via the actin-interacting protein NAB-1 (Chia et al., 2012, 2014). The angled geometry of the heterophilic SYG-1/SYG-2 complex was demonstrated to account for its synaptogenic properties, while rigidity of the adhesive complex allows close packing of SYG proteins, facilitating downstream signaling to the presynaptic cytoskeleton in *C.elegans* (Özkan et al., 2014). Since even poly-D-lysine-coated beads are able to induce presynaptic differentiation and local assembly of F-actin *in vitro*, it is suggested that cell adhesion by itself may be sufficient to induce F-actin assembly (Lucido et al., 2009), indicating the importance of cell-adhesion molecules in specifying the subcellular location of F-actin rearrangements. This is consistent with the emerging idea that various cell adhesion molecules often converge on a similar pathway that induces F-actin rearrangements (Nelson et al., 2013; Chua, 2014), which could ultimately lead to the capturing of SV proteins (Bury and Sabo, 2014) and thereby presynaptic assembly.

However, the notion of a role for actin in regulating presynaptic function in mature neurons is emerging (Rust and Maritzen, 2015). Actin is highly concentrated at synapses, and it was recently shown that axonal actin patches are localized to presynaptic sites and that F-actin accumulations become even more prominent when the neuron matures (D’Este et al., 2015). The formin-dependent dynamic F-actin filaments within the axon described earlier, is suggested to provide the delivery of F-actin to synapses, as attenuation of these actin trails by inhibition of formin decreases F-actin delivery to and intensity within presynaptic sites (Ganguly et al., 2015). However, within the presynaptic sites, actin is suggested to mainly exist in an Arp2/3-dependent branched network (Korobova and Svitkina, 2010; Figure 1D). Synaptic actin becomes even more enriched upon synaptic activity, suggesting additional recruitment and polymerization of actin at the presynapse (Sankaranarayanan et al., 2003). Despite its high abundance in mature synapses, the exact role of actin at the presynaptic site is currently ambiguous (Cingolani and Goda, 2008; Rust and Maritzen, 2015). Several roles have been suggested, such as a solely structural function, a role in recruiting and positioning of SVs, in regulating exocytosis or in controlling endocytosis (Halpain, 2003; Rust and Maritzen, 2015). In knock-out mice of the actin-depolymerizing proteins ADF/cofilin, the impairment of actin dynamics causes reduced SV recruitment to the AZ (Wolf et al., 2014). Here, actin may function in the recruitment and positioning of SVs by acting as a scaffold for the clustering of SVs via synapsin. Alternatively, actin may also provide the tracks that allow SV transport via actin-based motor proteins to the AZ or could be involved in fine-tuning the localization of SVs after endocytosis (Dillon and Goda, 2005; Cingolani and Goda, 2008; Rust and Maritzen, 2015). For long, it has been thought that actin could function as a barrier at the presynaptic membrane to control SV exocytosis (Dillon and Goda, 2005). However, this idea has been challenged by recent findings where increased F-actin levels, due to the absence of specific actin-depolymerizing proteins, led to increased exocytosis of SVs in mice (Wolf et al., 2014). Instead, this suggests that normal F-actin dynamics are essential for proper SV exocytosis (Rust and Maritzen, 2015), which could be, at least partially, mediated by the activity-dependent F-actin assembly function of the AZ protein Piccolo (Waites et al., 2011; Wagh et al., 2015). Defects in actin function or dysregulation of synaptic actin are also implicated in several mental disorders (Bernstein et al., 2011). Moreover, altered synapse development and morphology has been observed in a mouse model for Fragile X syndrome. These defects are presumably caused by dysregulation of actin dynamics, as altered levels of several actin-regulating proteins were observed (Klemmer et al., 2011). Moreover, mutant α-synuclein, associated with familial Parkinson’s disease (PD), was demonstrated to alter the rate of actin polymerization and to disrupt the actin cytoskeleton *in vitro* (Sousa et al., 2009).

Although actin has been regarded as the main cytoskeletal element within presynaptic sites, a possible role for MTs should also be noted (Figure 1D). Recently, the homolog of the microtubule stabilizing and bundling protein MAP1, Futsch, was demonstrated to localize to the neuromuscular junction (NMJ) terminals, where it regulates neurotransmitter release and AZ density in *Drosophila* (Lepicard et al., 2014). Here, Futsch localizes in between the AZ and MTs and thereby possibly links these two structures. Futsch might act in stabilizing and maintaining presynapse composition by reinforcing the interaction with the underlying microtubule cytoskeleton. In addition, microtubule reorganization via Futsch was demonstrated to be important in the activity-dependent remodeling of the AZ in *Drosophila* (Sugie et al., 2015). Prolonged exposure to environmental stimuli removes liprin-α, RIM-binding protein DRBP and Bruchpilot (Brp) from the AZ in sensory neurons, but not SYD-1 or Ca^2+^-channel Cacophopny (Cac). This altered localization of AZ components is suggested to occur via the divergent canonical Wnt signaling pathway, which affects presynaptic microtubule stabilization through phosphorylation of Futsch (Sugie et al., 2015). Moreover, it has been reported that during synapse remodeling, microtubule dynamic increases which is required for proper kinesin-1 mediated axonal transport of synaptic components (Kurup et al., 2015). Likewise, mammalian MAP1 has also been implicated in regulating the distribution of AZ components. Both MAP1A light chain 2 (MAP1A-LC2) and MAP1B-LC1 are reported to link N-type calcium channels to the actin cytoskeleton within the presynaptic site and to affect their degradation (Leenders et al., 2008; Gandini et al., 2014). More recently, MAP1B-LC1 was also found to interact with the presynaptic immunoglobulin protein KIRREL3 (Liu et al., 2015). These findings could establish exciting future research into the role of MTs in the organization and function of presynaptic sites.

### The Function of the Cytoskeleton in Axon Branching

The formation of axonal branches relies heavily on cytoskeleton rearrangements. Actin assembly initiates filopodia formation, whereas subsequent microtubule invasion is important for stabilizing the branch (Gentil and Cooper, 2012; Kalil and Dent, 2014). For example, disruption of F-actin dynamics in neurons leads to a loss of axonal branching *in vitro* and *in vivo*, while elongation of the axon remains unaffected (Dent and Kalil, 2001; Spillane et al., 2011). Since synapses are often present at axonal branch points, it is suggested that synapse formation might promote the development of axonal branches (Vaughn, 1989). Consistently, synapse and axonal branch formation was found to often occur simultaneously in zebrafish developing retinal ganglion cell (RGC) axons, linking these two processes (Meyer and Smith, 2006). More recently, a common molecular pathway that links synapse formation to branching was described in *C. elegans* (Chia et al., 2014). Here, the interaction of the cell adhesion molecules SYG1/2 initiates axon branching, besides initiating presynapse assembly as described before Chia et al. (2014). SYG-1 recruits the WVE-1/WAVE regulatory complex (WRC), an activator of the Arp2/3 complex, to synapses. This triggers assembly of an axonal F-actin patch required for both synapse assembly and axonal arborization (Chia et al., 2014). MTs that invade axonal filopodia show a high degree of debundling and seem to interact with F-actin (Ketschek et al., 2015). This microtubule debundling might promote the ability of MTs to target filopodia (Ketschek et al., 2015). Branch formation is promoted by nerve growth factor (NGF), which acts on the microtubule cytoskeleton and promotes the localized debundling of MTs along the axon (Ketschek et al., 2015).

### Axonal Transport

Besides its role in regulating and maintaining axonal polarization, outgrowth and stabilization, the axonal cytoskeleton also plays an essential role in active transport of axonal proteins, vesicles and organelles throughout the axon. Since the axon is functionally completely different from the dendrites, it requires a different set of proteins and cellular organelles. Active motor protein-driven transport is essential for sorting these axonal cargoes and to ensure that the correct proteins end up at the correct location within the cell (Kapitein and Hoogenraad, 2011). Active transport is especially important in axons, due to their significant length.

Three classes of motor proteins, kinesins, dyneins and myosins, transport cargoes along the cytoskeleton. Myosin moves specifically along actin filaments and is generally involved in contractile forces and short-range transport, while kinesin and dynein move along the microtubule cytoskeleton to facilitate long-range transport (Vale, 2003). Kinesin and dynein move in opposite directions: dynein moves towards the minus-end of MTs, whereas most kinesins move towards the plus-end (Hirokawa et al., 2010). Due to the unique microtubule organization in axons, kinesins are therefore responsible for the anterograde transport of axonal proteins while dynein enables retrograde transport (Kapitein and Hoogenraad, 2011; Figure 1A). Despite the broad variety of motor proteins, they share several general features. All motor proteins contain a relatively highly conserved motor domain, which associates with the cytoskeleton and binds ATP, needed for the generation of energy for movement. Motor proteins have a more diverse tail region, which associates with the cargo and contains several elements for regulation (Vale, 2003; Hirokawa et al., 2010). The diversity of the tail domain across various motor proteins allows for the ability of different motors to bind specific cargoes, whereby adaptor proteins further assist in establishing correct cargo-motor protein associations (Hirokawa and Takemura, 2005; Schlager and Hoogenraad, 2009).

Axonal transport is essential for the distribution of vesicles, organelles and signaling molecules along the axon to control polarization, axon elongation, and synapse function (Schlager and Hoogenraad, 2009; Chia et al., 2013; Maeder et al., 2014). A large proportion of these axonal cargoes is destined for the presynapse and needs to be delivered to these specialized sites through active transport (Goldstein et al., 2008; Hirokawa et al., 2010; Figure 1A). It has been suggested that several AZ proteins, including bassoon, piccolo and ELKS, are trafficked as preassembled complexes, whereas SV proteins and a distinct set of AZ proteins are transported by other types of vesicles (Shapira et al., 2003; Maas et al., 2012). However, this notion has become questionable since SV proteins and AZ proteins were reported to be co-trafficked, likely via heterogeneous multi-vesicle transport complexes (Tao-Cheng, 2007; Bury and Sabo, 2011; Wu et al., 2013).

Due to the heterogeneity of these vesicles and potential co-trafficking of various cargoes (Wu et al., 2013), the mechanism underlying active transport of these various synaptic components is not completely understood. It is known that anterograde transport of SV precursors is dependent on kinesin-3 family motors KIF1A and KIF1Bβ (Hall and Hedgecock, 1991; Okada et al., 1995; Kondo et al., 2012) via adaptor proteins like DENN/MADD or liprin-α (Hall and Hedgecock, 1991; Okada et al., 1995; Shin et al., 2003; Miller et al., 2005; Niwa et al., 2008). Another kinesin motor, KIF5, has also been linked to the transport of synaptic components. It is suggested that KIF5 specifically transports proteins destined for the presynaptic membrane, including syntaxin via the adaptor syntabulin, and SNAP25 (Diefenbach et al., 2002; Su et al., 2004; Cai et al., 2007; Goldstein et al., 2008; Niwa et al., 2008; Morton et al., 2010). Besides synaptic components, other proteins and organelles are also actively transported along the axon. Amyloid precursor protein (APP) vesicles and dense core vesicles (DCV), containing the neurotrophic factor brain-derived neurotrophic factor (BDNF), are both trafficked via KIF5 via the adaptor JIP1 and Huntington, respectively (Kamal et al., 2000; Colin et al., 2008; Lo et al., 2011; Fu and Holzbaur, 2013). The latter can also be trafficked by KIF1A (Lo et al., 2011). Mitochondria are transported by KIF5 (Tanaka et al., 1998; Pilling et al., 2006; Campbell et al., 2014) via the adaptors Miro1/2-TRAK1/2, syntabulin, RanBP2 or FEZ1 (Cai et al., 2005; Fransson et al., 2006; Fujita et al., 2007; Brickley and Stephenson, 2011; Patil et al., 2013; van Spronsen et al., 2013; Babic et al., 2015). There are also indications that other kinesins contribute to mitochondrial transport within the axon, including KIF1Bα (Nangaku et al., 1994) and Kinesin-like protein 6 (KLP6; Tanaka et al., 2011), to provide the synapses with the energy needed to meet their metabolic demand and to change synaptic energy levels and thereby synaptic activity (Sun et al., 2013).

Since most of the cargoes destined for the axon are made in the cell body, the axon needs to make use of some kind of mechanism that ensures that these cargoes are equally distributed throughout the axon and do not only accumulate at the most proximal sites. To overcome this challenge, a model is proposed in which inefficient capture of cargoes at synaptic sites and back-and-forth movements of cargoes enable cargoes to target synapses equally (Wong et al., 2012). This may be achieved by bidirectional or stop-and-go transport and by having reversible interactions of motor-cargo complexes with presynaptic sites. This view is supported by a biophysical model which suggests that a more democratic distribution of cargoes along the axon can indeed be achieved by making the transport process less efficient (Bressloff and Levien, 2015), implying that the activity and motility of kinesins needs to be correctly regulated to ensure proper cargo transport and delivery along the axon (Figure 1A).

The regulation of kinesin activity exists both at the level of kinesin-microtubule interactions and at the level of kinesin-cargo interactions. Distinct post-translational modifications of MTs are able to directly modulate the activity of specific kinesins, while leaving other kinesins unaffected (Janke and Bulinski, 2011; Song and Brady, 2015). Furthermore, MAPs are known to regulate transport by modulating the interaction of motors with the MTs (Vershinin et al., 2007; Dixit et al., 2008). In addition, the nucleotide-state of tubulin is suggested to affect the interaction of specific kinesins with MTs, thereby directing polarized transport due to the relative abundance of GTP-loaded MTs in the proximal part of the axon (Nakata et al., 2011; Morikawa et al., 2015). In addition to microtubule modifications, the organization and spacing of MTs affects kinesin motility (Conway et al., 2014; Wortman et al., 2014; Stephan et al., 2015), whereas the dynamics of the MTs may influences the kinesin-microtubule interaction (Kurup et al., 2015). At the level of kinesin-cargo interactions, adaptor proteins direct the coupling of the cargo to the motor protein and function as a modulator of kinesin activity and motility (Akhmanova and Hammer, 2010; Maday et al., 2014). In addition to adaptor proteins, other mechanisms known to regulate cargo-motor associations include local Ca^2+^ concentrations, phosphorylation of kinesin motors and Rab-GTPase activity (Schlager and Hoogenraad, 2009). Besides these more subtle regulatory mechanisms of kinesin activity, kinesins can also be completely inhibited by the mechanism of autoinhibition. Here, the kinesin folds back on itself, enabling the tail-domain to bind and inhibit its own motor-domain (Verhey and Hammond, 2009). Although this mechanism is best described for kinesin-1 motor KIF5 (Verhey et al., 1998; Kaan et al., 2011), members of other kinesin families including KIF1A (Lee et al., 2004; Hammond et al., 2009), KIF17 (Hammond et al., 2010) KIF13B (Yamada et al., 2007), KIF21A (van der Vaart et al., 2013) and KIF16B (Farkhondeh et al., 2015) are also known to be regulated by autoinhibition. This inhibition can be released, and thereby regulated, by cargo binding or phosphorylation of the kinesin (Verhey and Hammond, 2009).

Impairments in axonal transport, caused by dysfunctioning or dysregulation of motor proteins, regulatory proteins or the underlying cytoskeleton could all have severe consequences for axonal functioning (Tischfield et al., 2011; Millecamps and Julien, 2013; Niwa et al., 2013; Encalada and Goldstein, 2014). Indeed, an increasing amount of neurodegenerative disorders is linked to abnormalities in transport-related proteins (De Vos et al., 2008; Hirokawa et al., 2010). For example, mutations in KIF5A have been linked to HSP and Charot-Marie-Tooth disease type 2 (CMT2; Liu et al., 2014). Likewise, mutations in the microtubule growth inhibiting kinesin KIF21A contribute to congenital fibrosis of the extraocular muscles type 1 (CFEOM1; Yamada et al., 2003), due to loss of autoinhibition of the microtubule growth inhibitor KIF21A (Cheng et al., 2014). These are only one of the few examples of the many transport-related neurological disorders, highlighting the importance of correct axonal transport functioning and regulation for proper axonal function.

## Conclusions and Outlook

The axon is unique in its morphology and function and it contains several specialized structures and mechanisms that ensure proper axonal functioning. Here, we focused on the cytoskeletal network within the axon and discussed how the axonal cytoskeleton contributes to the function of these various structures and processes. Identifying the organization of both the microtubule and actin cytoskeleton within the axon shaft, AIS and presynaptic sites has increased our understanding of their contribution to axon functioning. However, comprehensive knowledge on the how the cytoskeleton organization relates to the function of specific axonal structures is limited. Therefore, it will be important to elucidate the currently ambiguous role of actin within the presynapse to understand the molecular mechanisms of presynaptic organization and functioning. Also, it will be important to examine the contribution of MTs in presynaptic functioning. In addition, unraveling the molecular mechanisms of the barrier function of the AIS and how the underlying cytoskeleton contributes to this function will increase our understanding of the mechanisms of polarized transport. Moreover, further identification of motor proteins, their adaptors, cargoes, and regulatory mechanisms will be essential to understand the precise molecular mechanisms underlying axonal transport. Since an increasing numbers of neurodevelopmental and neurodegenerative diseases are being linked to defects in the axonal transport machinery, fundamental knowledge about intracellular transport mechanisms and cytoskeleton organization will be important for the development of new therapeutic strategies.

## Conflict of Interest Statement

The authors declare that the research was conducted in the absence of any commercial or financial relationships that could be construed as a potential conflict of interest.
